# Consensus from the Brazilian Academy of Neurology for the diagnosis, genetic counseling, and use of disease-modifying therapies in 5q spinal muscular atrophy

**DOI:** 10.1055/s-0044-1779503

**Published:** 2024-02-05

**Authors:** Edmar Zanoteli, Alexandra Prufer de Queiróz Campos Araujo, Michele Michelin Becker, Clarisse Pereira Dias Drumond Fortes, Marcondes Cavalcante França, Marcela Camara Machado-Costa, Wilson Marques, Ciro Matsui Jr, Rodrigo Holanda Mendonça, Flávia Nardes, Acary Souza Bulle Oliveira, Andre Luis Santos Pessoa, Jonas Alex Morales Saute, Paulo Sgobbi, Hélio Van der Linden, Juliana Gurgel-Giannetti

**Affiliations:** 1 Universidade de São Paulo, Faculdade de Medicina, Departamento de Neurologia, São Paulo SP, Brazil.; 2 Universidade Federal do Rio de Janeiro, Faculdade de Medicina, Departamento de Pediatria, Rio de Janeiro RJ, Brazil.; 3 Hospital de Clínicas de Porto Alegre, Departamento de Pediatria, Unidade de Neurologia Infantil, Porto Alegre RS, Brazil.; 4 Universidade Federal do Rio de Janeiro, Instituto de Puericultura e Pediatria Martagão Gesteira, Rio de Janeiro RJ, Brazil.; 5 Universidade Estadual de Campinas, Faculdade de Ciências Médicas, Departamento de Neurologia, Campinas SP, Brazil.; 6 Escola Bahiana de Medicina e Saúde Pública, Salvador BA, Brazil.; 7 Universidade de São Paulo, Faculdade de Medicina de Ribeirão Preto, Departamento de Neurociências e Ciências do Comportamento, Ribeirão Preto SP, Brazil.; 8 Universidade Federal de São Paulo, Departamento de Neurologia e Neurocirurgia, São Paulo SP, Brazil.; 9 Universidade Estadual do Ceará, Hospital Infantil Albert Sabin, Fortaleza CE, Brazil.; 10 Universidade Federal do Rio Grande do Sul, Faculdade de Medicina, Hospital de Clínicas de Porto Alegre, Serviços de Genética Médica e de Neurologia, Porto Alegre RS, Brazil.; 11 Centro de Reabilitação Dr. Henrique Santillo, Serviço de Neurologia Infantil e Neurofisiologia, Goiânia GO, Brazil.; 12 Universidade Federal de Minas Gerais, Faculdade de Medicina, Departamento de Pediatria, Belo Horizonte MG, Brazil.

**Keywords:** Spinal Muscular Atrophy, Survival of Motor Neuron 1 Protein, Oligonucleotides, Risdiplam, Genetic Therapy, Onasemnogene abeparvovec, Atrofia Muscular Espinhal, Proteína 1 de Sobrevivência do Neurônio Motor, Oligonucleotídeos, Risdiplam, Terapia Genética, Onasemnogene abeparvovec

## Abstract

Spinal muscular atrophy linked to chromosome 5 (SMA-5q) is an autosomal recessive genetic disease caused by mutations in the *SMN1* . SMA-5q is characterized by progressive degeneration of the spinal cord and bulbar motor neurons, causing severe motor and respiratory impairment with reduced survival, especially in its more severe clinical forms. In recent years, highly effective disease-modifying therapies have emerged, either acting by regulating the splicing of exon 7 of the *SMN2* gene or adding a copy of the *SMN1* gene through gene therapy, providing a drastic change in the natural history of the disease. In this way, developing therapeutic guides and expert consensus becomes essential to direct the use of these therapies in clinical practice. This consensus, prepared by Brazilian experts, aimed to review the main available disease-modifying therapies, critically analyze the results of clinical studies, and provide recommendations for their use in clinical practice for patients with SMA-5q. This consensus also addresses aspects related to diagnosis, genetic counseling, and follow-up of patients under drug treatment. Thus, this consensus provides valuable information regarding the current management of SMA-5q, helping therapeutic decisions in clinical practice and promoting additional gains in outcomes.

## Resumo

Atrofia muscular espinhal ligada ao cromossomo 5 (AME-5q) é uma doença genética de herança autossômica recessiva causada por mutações no gene *SMN1* . A AME-5q cursa com degeneração progressiva dos motoneurônios medulares e bulbares, acarretando grave comprometimento motor e respiratório com redução da sobrevida, especialmente nas suas formas clínicas mais graves. Nos últimos anos, terapias modificadoras da doença altamente eficazes, ou que atuam regulando o *splicing* do exon 7 do gene *SMN2* ou adicionando uma cópia do gene *SMN1* via terapia gênica, têm surgido, proporcionando uma mudança drástica na história natural da doença. Dessa forma, o desenvolvimento de guias terapêuticos e de consensos de especialistas torna-se importante no sentido de direcionar o uso dessas terapias na prática clínica. Este consenso, preparado por especialistas brasileiros, teve como objetivos revisar as principais terapias modificadoras de doença disponíveis, analisar criticamente os resultados dos estudos clínicos dessas terapias e prover recomendações para seu uso na prática clínica para pacientes com AME-5q. Aspectos relativos ao diagnóstico, aconselhamento genético e seguimento dos pacientes em uso das terapias também são abordados nesse consenso. Assim, esse consenso promove valiosas informações a respeito do manejo atual da AME-5q auxiliando decisões terapêuticas na prática clínica e promovendo ganhos adicionais nos desfechos finais.

## INTRODUCTION

Spinal muscular atrophy (SMA) is a group of diseases characterized by degeneration of motor neurons in the spinal cord and brainstem leading to muscle weakness. The most common form of SMA is caused by mutations in the survival motor neuron 1 ( *SMN1* ) gene located at 5q13 (SMA-5q). 1 SMA-5q has an autosomal recessive inheritance, and its global incidence is estimated at 1 in 10,000 live births. 2 3 Despite being classified as a rare disease, SMA-5q leads to high infant mortality and significant family, social, and economic impact. 2 3

The SMN protein is encoded by two genes, *SMN1* and its homologous gene *SMN2* , both located on chromosome 5. 1 4 5 6 In approximately 96% of SMA-5q patients, the disease is caused by a homozygous deletion of exons 7 and 8 of the *SMN1* , or in some cases, just exon 7. 1 In about 4% of cases, there is a deletion in one allele and a point mutation in the other allele. The carrier frequency of genetic alterations in the *SMN1* varies among different populations, from 1 in 38 to 1 in 72. 7 8

The *SMN2* differs from *SMN1* by a single nucleotide variant (840C → T) in exon 7, resulting in the loss of exon 7 from most transcripts during messenger RNA processing, leading to the translation of a truncated and unstable SMN protein. 1 The *SMN2* can generate only 10% to 20% of functioning SMN protein. 5 6 The number of copies of the *SMN2* acts as a phenotype modifier, meaning the more copies of *SMN2* a patient has, the less severe the clinical phenotype tends to be. 1 4 9 Table 1 defines the different clinical phenotypes or levels of severity, with age at onset of symptoms and maximum motor milestone reached and the correlation with the number of copies of the *SMN2* .

**Table 1:** SMA-5q clinical classification and correlation with the number of copies of the *SMN2* gene

SMA type	Prevalence	Symptoms onset	Maximum motor ability	Subclassification	*SMN2* copy number
Type 0	Unknown	Pre-natal	None	–	1 copy
Type 1	60%	0-6 months	Hold the head	1A: start < 1 month. 1B: Start in 1-3 months. 1C: Start in 3-6 months.	1-2 copies
Type 2	27%	7-18 months	Sit	2A: Sit without support but lose this capacity. 2B: Sit without support but maintain this capacity.	3 copies in more than 70% of patients
Type 3	12%	> 18 months	Walk	3A: Start in 18-36 months. 3B: Start after 36 months.	3-4 copies in more than 95% of patients
Type 4	1%	> 18 years	Walk		4 or more copies in more than 90% of patients

Various therapeutic approaches are currently being developed for SMA-5q, including SMN-dependent and SMN-independent therapies. SMN-dependent therapies focus on addressing the SMN protein deficiency, such as gene therapy with *SNM1* gene replacement (onasemnogene abeparvovec-AVXS101), the inclusion of exon 7 in *SMN2* (nusinersen, risdiplam), and upregulation of the *SMN2* transcript (salbutamol, celecoxib, hydroxyurea). 10 Some of these therapies have already been approved by the leading international regulatory agencies and the Brazilian National Surveillance Agency (ANVISA).

This consensus, prepared by Brazilian specialists in SMA-5q treatment, aims to review the main disease-modifying drug therapies available, critically analyze their results, and provide recommendations for their use in SMA-5q. The consensus provides valuable insights into the current management of SMA-5q and helps guide treatment decisions to improve patient outcomes.

## METHODS

The consensus on SMA-5q was developed using evidence-based recommendations by a group of 16 specialists in neuromuscular disorders and or members of the Neuromuscular Disorders Department of the Brazilian Academy of Neurology. All participants are neurologists or child neurologists with experience in SMA-5q.

The consensus-building process began with an online meeting where all members discussed and agreed on the topics to be addressed. The selected topics included genetic diagnosis, disease modifiers therapies (nusinersen, risdiplam, gene replacement therapy), follow-up of SMA-5q patients, and genetic counseling. Subsequently, the participants were divided into working groups based on their chosen topic. Each working group performed a systematic search of the databases Medline (PubMed), Scopus, Web of Science, and the Cochrane Library from 2010 through 2023 to include more recent articles related to the use of disease-modifying therapies and genetic diagnosis. The keywords used in the search were a combination of “Atrophy, Spinal Muscular” with any one of the following alone or in combination: “Gene replacement therapy”, “Onasemnogene abeparvovec”, “AVXS101”, “Nusinersen”, “Risdiplam”, “Treatment”, “Genetic diagnosis”, “Follow up”, and “Genetic counseling”. The working groups excluded case reports, narrative reviews, and expert opinions, focusing on studies with higher levels of evidence. 11

After the literature review, a modified Delphi method was chosen to achieve a consensus on the recommendations of the group of specialists. 12 The statements developed by each working group were submitted to all members on an anonymous voting system, using a Google form platform, of five different Likert scale options: 1. strongly agree; 2. agree; 3. neither agree nor disagree; 4. disagree; and 5. strongly disagree. All members responded to the questionnaire, and when more than 80% of the participants agreed, the consensus was considered valid and very strong. For the questions with less than 80% agreement, we invited the coordinators of each group to debate and reformulate them. After that, all participants answered the questions in a second round, and a consensus of 80% agreement was achieved for all questions. Based on these questions, we built the consensus recommendations.

## RESULTS

### Genetic diagnosis for SMA-5q

Chart 1 provides a summary of recommendations for the genetic diagnosis of SMA-5q. SMA-5q diagnosis is primarily based on demonstrating biallelic pathogenic variants in the *SMN1* . 13 The initial tests should focus on searching for *SMN1* deletions, which can be achieved using molecular techniques such as quantitative PCR (qPCR) or multiplex ligation-dependent probe amplification (MLPA). 14 15 16 17 Next-generation sequencing (NGS) has also enabled the detection of *SMN1* deletion. 18 Standard PCR with restriction digest is another method but cannot quantify *SMN2* copy number or detect compound heterozygous status. 19 It is crucial to highlight that gene-targeted microarray is not helpful in suspected SMA-5q cases due to the similarity between *SMN1* and *SMN2* . 20

**Chart 1:** Recommendations for the genetic diagnosis of SMA-5q

Confirmation of biallelic pathogenic variant in *SMN1* gene	To establish a genetic diagnosis of SMA-5q, it is essential to demonstrate the presence of a biallelic pathogenic variant in the *SMN1* gene
MLPA technique for detection of deletion in exons 7 and 8 of *SMN1*	The MLPA technique is a reliable method for detecting deletions in specific exons of the *SMN1* gene, particularly exons 7 and 8. Additionally, MLPA can also be used to determine the number of copies of the *SMN2* gene, which is a related gene that influences the severity of SMA
Sequencing of *SMN1* gene for atypical cases	In cases where individuals show clinical symptoms of SMA but only demonstrate deletion in one copy of the *SMN1* gene (heterozygous deletion), or no deletion at all (especially in cases of consanguineous parents), further testing is necessary. For such cases, sequencing of the entire coding region of the *SMN1* gene and splicing regions located at the intron-exon junction should be performed. This search aims to identify small-scale pathogenic variants in one or both alleles that might not be detected by the MLPA technique
Genetic testing for babies born to mothers with SMA history	Newborn babies born to mothers with a previous history of other children diagnosed with SMA should undergo genetic testing at birth. Early genetic testing allows for early detection and intervention if SMA is confirmed
Investigation of other neuromuscular causes	In cases where individuals present clinical symptoms of SMA-5q, but no deletion is detected in the *SMN1* gene and/or the sequencing of the entire coding region of the *SMN1* gene appears normal, it is important to investigate other potential neuromuscular causes
Newborn screening for SMA	Neonatal screening for SMA is an effective strategy to identify affected individuals in pre-symptomatic stage or during the early stage of clinical impairment

If no copy of exon 7 of *SMN1* is found, the diagnosis of SMA-5q is confirmed. However, if a single allele deletion is identified, additional testing, such as Sanger or NGS, should be pursued to check for point mutations in the other allele. Rare cases of SMA-5q caused by biallelic *SMN1* point mutations have been reported, 21 so *SMN1* sequencing should be considered, especially in patients born from consanguineous parents. In addition, the evaluation of *SMN2* copy number should always be included in the diagnostic work-up of SMA-5q.

No additional work-up beyond genetic testing is typically required for diagnosing SMA-5q, especially in subjects with a typical phenotype and or early onset (in the first few months of life). However, in cases where the phenotype is atypical, nerve conduction studies, needle electromyography, and muscle biopsy may help in the differential diagnosis between SMA-5q and other neuromuscular disorders such as muscle diseases. Serum creatine kinase (CK) levels might also be helpful, but it should be noted that mild elevations can be observed in SMA-5q. 13

### Treatment of SMA-5q with nusinersen

Chart 2 summarizes recommendations for the treatment of SMA-5q with nusinersen. Nusinersen (Spinraza®) is an antisense oligonucleotide (ASO) that targets an intronic splicing silencer site within the *SMN2* pre-messenger RNA downstream of exon 7. 22 This targeting of the splicing silencer allows for increased inclusion of exon 7 during mRNA processing, producing more functional SMN protein from the *SMN2* gene. As ASOs do not cross the blood-brain barrier, nusinersen must be administered via the intrathecal route to exert its therapeutic effect within the central nervous system. Nusinersen has a long half-life of approximately five months, which allows for less frequent dosing. The treatment regimen typically involves an initial loading phase, consisting of four loading doses administered over two months, followed by maintenance doses every four months. It is essential to adhere to the dosing schedule to maintain the therapeutic benefit. The dosage of nusinersen remains the same across all age groups.

**Chart 2:** Recommendations for the treatment of SMA-5q with nusinersen

Efficacy and safety in pre-symptomatic and SMA types 1, 2, and 3	Nusinersen has been shown to be effective and safe for the treatment of patients in the pre-symptomatic stage and those with SMA types 1, 2, and 3
Limited data for SMA types 0 and 4	The effectiveness and safety of nusinersen for patients with SMA types 0 and 4 are not yet well-established due to limited data
Less favorable outcome in severe motor and respiratory impairment	Patients with severe motor and respiratory impairment may have minor expectations for benefits with nusinersen treatment. The response to treatment may be influenced by the severity of the disease, irreversible loss of motor neurons, and associated complications
Contraindications for nusinersen	Nusinersen is contraindicated in patients at risk of repeated intrathecal administration, which includes conditions such as intracranial hypertension, hydrocephalus, and spinal dysraphism
Special considerations for severe scoliosis or previous spine surgery	In patients with severe scoliosis or a history of spine surgery, the administration of nusinersen should be performed by an experienced healthcare professional. Additionally, the use of auxiliary imaging methods like ultrasound, tomography, or fluoroscopy may be necessary to ensure safe and accurate delivery of the drug
Regular monitoring for adverse events	Patients receiving nusinersen treatment should be regularly monitored for potential adverse events. Monitoring every 6 months for urine protein, platelets, and renal function is recommended. Depending on the occurrence of adverse events, some patients may require more frequent testing

The initial human clinical phase 1 and 2 studies of nusinersen in children with SMA type 1 and types 2 and 3 were published in 2016. 22 23 These studies provided valuable insights into the safety and efficacy of nusinersen in different types of SMA. The ENDEAR, a placebo-randomized clinical trial, demonstrated significant benefits of nusinersen treatment in infants with SMA type 1 (< 7 months). 24 The trial showed that nusinersen-treated patients had more prolonged survival and more significant improvements in motor function compared to those without treatment.

Subsequent real-life studies with type 1 SMA patients, including those using the drug in an expanded access program, further confirmed the positive effects of nusinersen on motor and respiratory function, as well as on the survival of patients with long-term illness and varying respiratory conditions. 25 26 27 28 29 30 31 32 33 34 35 36 37 38 39 40

The CHERISH trial was a placebo-randomized study that enrolled 126 patients with late-onset SMA aged 2 to 12 years. 41 The study demonstrated that patients who received nusinersen experienced clinically significant improvements in motor function compared to the control group. Motor function improvement was measured using the Hammersmith Functional Motor Scale Expanded (HFMSE) scale, a commonly used tool to assess motor abilities in SMA patients. Post hoc analyses conducted on children from the CHERISH study showed a greater response of nusinersen in those with absence or mild scoliosis at baseline. 42

Subsequent real-life studies, including both prospective and retrospective observational studies, have further supported the beneficial effects of nusinersen on motor function in pediatric and adult patients with SMA types 1, 2, and 3. 40 41 42 43 44 45 46 47 48 49 50 51 52 53 54 55 56 57 58 59 60 61

The CS2 phase 1/2 study, followed by the CS12 phase 2, an open-label extension study, demonstrated long-term benefits of nusinersen in patients with SMA types 2 and 3, aged 2–15 years at enrollment. 62 The follow-up of approximately three years showed improvements in motor function and stabilization of disease activity. In the CS3A study, which focused on patients with SMA type 1, the final analysis demonstrated a durable clinical response in a significant proportion of the treatment cohort. 63 The results were comparable to those seen in the ENDEAR study, which showed significant improvements in motor function and survival in infants with SMA type 1 who received nusinersen. The median follow-up in the CS3A study was 36.2 months, and 75% of the participants were still alive at the time of study closure. These findings indicate that nusinersen has the potential to provide meaningful and long-lasting clinical benefits for patients with SMA type 1.

Meta-analysis studies have confirmed the efficacy and safety of nusinersen for children and adult patients with SMA and indicate that more randomized clinical trials would be ideal for increasing the level of evidence. 64 65 66 A systematic review and meta-analysis of adverse events in treating SMA with nusinersen was published recently. 67 Data from 969 children and adolescents was analyzed. The overall rate of adverse events was 83.51%, and serious adverse events were 33.04%. Fever was the most common specific adverse event, followed by upper respiratory tract infection and pneumonia. The incidence of severe and fatal adverse events was significantly lower than in the placebo group. 67

A higher dose of nusinersen is being tested in patients with early and late-onset SMA (DEVOTE study). 68 In Part A, all six enrolled participants aged 6.1 to 12.6 years have completed the study. Common adverse events (headache, pain, chills, vomiting, and paresthesia) were considered related to the lumbar puncture procedure, and there were no safety concerns regarding clinical or laboratory parameters. Most participants showed stabilization or improved motor function. 68 Parts B and C of the DEVOTE are ongoing.

### Pre-symptomatic treatment of SMA-5q with nusinersen

NURTURE (CS5) is an ongoing phase 2, open-label, single-arm, multinational study that has demonstrated the potential of early intervention with nusinersen in pre-symptomatic infants with either 2 or 3 *SMN2* copies. 69 With a median follow-up of 2.9 years, the infants in the study (median of 34.8 months of age) had surpassed the expected age of symptom onset for SMA types 1 or 2, and all of them were alive without the need for tracheostomy or permanent ventilation. While a small proportion (16%) of participants with two *SMN2* copies required respiratory support during acute, reversible illnesses, the vast majority did not need permanent ventilation. Almost all the participants achieved the ability to sit without support (92%), and the majority achieved walking with assistance or independently (88%).

A recent paper showed the continued benefit of the nusinersen in pre-symptomatic patients followed for five years of treatment (NURTURE study). 70 All patients were alive, and none discontinued the treatment or utilized respiratory intervention. Children with three *SMN2* copies achieved all WHO motor milestones, and all children with two *SMN2* copies achieved sitting without support, 4/15 walking with assistance, and 13/15 walking alone. 70

### Safety of nusinersen

Lumbar puncture, while a common medical procedure, carries several potential complications, including post-lumbar puncture headache, vomiting, back pain, bleeding, and infection. 65 In clinical studies of nusinersen, fewer severe adverse events were observed when compared to the control group as well as events leading to treatment discontinuation. 24 41 65 Common adverse events associated with nusinersen were pyrexia, upper respiratory tract infections, and constipation. 24 41 65

### Treatment of SMA-5q with risdiplam

Chart 3 provides a summary of recommendations for the treatment of SMA-5q with Risdiplam. Risdiplam (Evrysdi®) is a small oral molecule designed to selectively modify the splicing of *SMN2* pre-mRNA and promote the inclusion of exon 7 to increase levels of functional SMN protein from a complete mRNA transcript. 71 Risdiplam has been approved in more than 80 countries worldwide. Registration approval in Brazil occurred in October 2020, and insertion in the public care system in 2022.

**Chart 3:** Recommendations for the treatment of SMA-5q with risdiplam

Efficacy and safety in pre-symptomatic and SMA types 1, 2, and 3	Risdiplam has been shown to be effective and safe for the treatment of patients in pre-symptomatic stage and those with SMA types 1, 2, and 3
Limited data for SMA types 0 and 4	The effectiveness and safety of risdiplam for patients with SMA types 0 and 4 are not well-established due to limited data
Long-term safety and age considerations	Additional studies are required to evaluate the long-term safety and efficacy of risdiplam, especially for individuals over 25 years of age. The safety evidence for risdiplam, based on scientific publications, currently spans up to 4 years
Less favorable outcome in severe motor and respiratory impairment	Patients with severe motor and respiratory impairment may have minor expectations for the benefits of risdiplam treatment. The response to treatment may be influenced by the severity of the disease, irreversible loss of motor neurons, and associated complications
Laboratory follow-up	Although general laboratory abnormalities are not frequently reported in patients taking risdiplam, follow-up monitoring every 6 months for urine protein, renal function, and serum platelets and liver enzymes is recommended

The FIREFISH study is investigating the safety and efficacy of risdiplam in treated infants with type 1 SMA versus historical controls. 72 73 The dose currently in use was defined in part 1 of the study. Part 2 of the study analyzed 41 infants with SMA type 1 (1-7 months) with two *SMN2* copies. In part 2, after 12 months of treatment, infants with type 1 SMA could sit without support for at least 5 seconds. 73 After 24 months of treatment, 38 infants were ongoing in the study, and 18 infants (44%) were sitting without support for at least 30 seconds. 74 Seven more infants achieved head control from month 12 to month 24. No infant could stand alone or walk alone after 24 months of treatment. The event-free survival at month 24 was 34 of 41 infants (83%) versus 35 infants (85%) at month 12. The most frequently reported adverse event was upper respiratory tract infection in 22 infants (54%); the most common serious adverse events were pneumonia in 16 infants (39%) and respiratory distress in three infants (7%). 74 Although not yet published, the latest data from the FIREFISH study presented at the 2023 Annual SMA Conference showed that after four years of treatment, 91% of infants treated were still alive, and 64% of them were able to sit for at least 5 seconds, and 96% maintained the swallowing capacity. 75 Furthermore, the study also showed continued reductions in serious adverse events and hospitalizations over time.

SUNFISH, a phase 3, randomized, double-blind, placebo-controlled study, investigates the efficacy and safety of risdiplam in type 2 and non-ambulant type 3 SMA aged 2 to 25 years. 76 Part 1 of the study showed that a median two-fold increase of serum SMN protein was obtained within four weeks of treatment initiation at the highest dose level. This increase in SMN protein was sustained over 24 months of treatment. 76 Part 2 is the randomized, double-blind, placebo-controlled portion of the SUNFISH study, which included 180 patients with SMA type 2 or non-ambulatory type 3. Randomization was stratified by age group (2 to 5 years, 6 to 11 years, 12 to 17 years, 18 to 25 years). In part 2 of the study, the primary endpoint was met: a significantly greater change from baseline in the 32-item Motor Function Measure (MFM32) total score was observed with risdiplam compared with placebo at month 12. 77 At month 24 of risdiplam treatment, 32% of patients demonstrated improvement with a gain of three or more points from baseline in MFM32 total score; while 58% of the treated patients experienced stabilization or improvement (change of ≥ 0), confirming the benefit of longer-term treatment. 78 After four years of treatment, the change from baseline in the MFM32 total score was generally stable. 79 In addition, patients and caregivers reported continued improvement or stabilization in the level of help needed for activities of daily living based on the SMAIS-ULM scale. The safety profile after 48 months was consistent with that observed after 12 months. 79

The JEWELFISH is an ongoing, multicenter, open-label study designed to assess the safety, tolerability, pharmacokinetics, and pharmacodynamics of risdiplam in the broadest population ever studied in an SMA trial, including patients with types 1 to 3 SMA (n = 174) with a wide range of ages (1 to 60 years), disease severities, and who have previously received other disease-modifying therapies (RG7800,7 nusinersen, olesoxime or onasemnogene abeparvovec). 80 The JEWELFISH study population had a similar safety profile and increase in SMN protein levels after 12 months of treatment with risdiplam compared with treatment-naïve patients who were treated with risdiplam in the FIREFISH and SUNFISH clinical trials. 80 An increase in the total distance walked in the 6MWT was observed (median 30.88 meters) in ambulant patients over 24 months of treatment with risdiplam. 80 No safety concerns were observed in the included patients after 24 months of treatment with risdiplam.

Real-world experience with risdiplam has also been published and further supported its beneficial effects on motor function in patients with SMA. 81 82 83 84 A group of 155 SMA type 1 and 2 patients (26 naive of drug modifying therapy), most in age groups of 6 to 18 years, 52.9% type 2, and 149 non-ambulant were granted the opportunity to use risdiplam through an expanded access program in the US. 82 The results were similar to the pivotal studies. Additionally, similar results were found in a short-term follow-up compassion study in Germany, including SMA types 1 and 2. 81 A more recent publication of a systematic review and metanalysis study reported that after 12 months of treatment with risdiplam, 57% of participants with SMA type 1 achieved a CHOP-INTEND score ≥ 40 points, and more than half were able to feed orally and had head control. 85 In SMA types 2 and 3, MFM32, RULM, and HFMSE increased by 2.09, 1.73, and 1.00 points, respectively. 85

### Pre-symptomatic SMA-5q treatment with risdiplam

RAINBOWFISH is an ongoing, multicenter, open-label, single-arm study to assess the efficacy and safety of risdiplam in infants with genetically diagnosed pre-symptomatic SMA-5q. Preliminary data showed that most infants treated with risdiplam could sit independently, and many were standing and walking as assessed by the Hammersmith Infant Neurological Examination-2 (HINE-2) at month 12. 86 After 12 months of treatment, most infants achieved near-maximum CHOP-INTEND total scores. All infants maintained bulbar function, and none required permanent ventilation after 12 months of treatment. 86 No serious adverse effects were reported.

### Safety of risdiplam

Risdiplam has shown an overall good safety profile in clinical studies (FIREFISH, SUNFISH, JEWELFISH, RAINBOWFISH). Preclinical retinal toxicity has demanded careful follow-up during those trials; however, no ophthalmologic toxicity was detected in humans using a therapeutic dose. 87 Drug-to-drug interaction, using midazolam with and without risdiplam in adults and children, found negligible CYP3A inhibition risk. 88 Furthermore, the effect of mild or moderate hepatic impairment on the plasma pharmacokinetics of a single dose of risdiplam was compared with matched subjects with normal hepatic function, and results show that no dosing adjustment is needed under those circumstances. 89 Gastrointestinal disorders have been reported in risdiplam-treated patients. 81 82

### Treatment of SMA-5q with gene replacement therapy (onasemnogene abeparvovec)

Chart 4 summarizes recommendations for the treatment of SMA-5q with onasemnogene abeparvovec. Onasemnogene abeparvovec (OAV101 or Zolgensma®) is a gene replacement therapy based on a self-complementary adeno-associated virus serotype 9 (AAV9) vector that carries a functional copy of the human *SMN1* gene. 90 This gene replacement therapy aims to provide continuous expression of SMN protein in the body, effectively addressing the underlying cause of SMA-5q. The administration of onasemnogene abeparvovec is performed intravenously, allowing the AAV9 vector to cross the blood-brain barrier and target neurons in the central nervous system.

**Chart 4:** Recommendations for the treatment of SMA-5q with gene replacement therapy with onasemnogene abeparvovec

Efficacy for pre-symptomatic and SMA type 1	Gene replacement therapy has shown to be effective for patients in pre-symptomatic stage and those with SMA type 1, up to 6 months old
Efficacy for SMA types 1 and 2, up to 24 months	Gene therapy is considered effective for patients with SMA types 1 and 2, aged up to 24 months, with a body weight of up to 13.5 kg
Favorable risk/benefit ratio	The risk/benefit ratio of gene therapy treatment is favorable for individuals in pre-symptomatic stage of SMA and for patients with SMA types 1 and 2 up to 24 months of age, with a body weight of up to 13.5 kg. However, it is important to note that the occurrence of complications related to gene therapy may increase with increasing weight and age of the child
Limited evidence for older patients	There is insufficient evidence of the efficacy and safety of gene replacement therapy for patients with SMA types 1, 2, or 3, aged over 24 months (even with a body weight of up to 13.5 kg), or of any age but with a body weight of over 13.5 kg. Further research is needed to evaluate the benefits and risks of gene therapy in these populations
Anti-AAV9 antibody dosage	Prior to gene therapy administration, patients should undergo anti-AAV9 antibody dosage. The administration of gene therapy is contraindicated when the anti-AAV9 titer is greater than 1:50 measured within 30 days before the therapy
Corticosteroid treatment	Corticosteroid treatment should accompany gene therapy administration to reduce the risk of immune responses. A dosage of 1 to 2 mg/kg/day should be started one day before administration and maintained for at least 1 month, with a slow dose reduction over another month, or based on the transaminase levels
Laboratory monitoring: regular laboratory monitoring is crucial during gene therapy treatment	This includes complete blood count, coagulogram, renal function, transaminases, and troponin measurements before administration and at specific intervals during and after corticosteroid use
Persistent abnormalities in transaminase levels	If patients show persistent abnormalities in transaminase levels, further investigation, and prolonged use of corticotherapy may be required to prevent liver failure
Hepatologist evaluation	Patients who have elevated transaminases and do not adequately respond to corticosteroid treatment at a dose of 1 mg/kg/day should be promptly evaluated by a hepatologist.
Hospital administration	Gene therapy should be administered in a hospital environment capable of managing possible complications
Clinical stability and infections	Patients should be clinically stable in their general health prior to gene therapy infusion. Treatment should be delayed in patients with infections until the infection has resolved and the patient is clinically stable, for at least 2 weeks prior to infusion

The regulatory approvals for onasemnogene abeparvovec vary slightly between different regions. In the United States, the Food and Drug Administration (FDA) has approved it for the treatment of children with SMA-5q who are under two years of age. In Europe, the European Medicines Agency (EMA) indicates its use for children (up to 21 Kg) with bi-allelic *SMN1* gene mutations and a clinical diagnosis of SMA type 1 or children with up to three *SMN2* copies. In Brazil, the ANVISA has approved onasemnogene abeparvovec for babies younger than two years old and up to three copies of *SMN2* .

The START study was a pivotal clinical trial that evaluated the safety and efficacy of onasemnogene abeparvovec in patients with type 1 SMA who had two copies of the *SMN2* . 90 The trial included two cohorts of patients: a low-dose group with only three patients (mean age of 6.3 months) and a high-dose group with 12 patients (mean age of 3.4 months). At 20 months following the gene transfer, eleven of the 12 children receiving the high dose of gene therapy could sit unassisted and feed unassisted. 90 Data from the extension study showed maintenance of the effectiveness for at least five years. 91

The phase 3 studies STR1VE-EU conducted in Europe and STR1VE-US conducted in the USA further confirmed the effectiveness of onasemnogene abeparvovec in treating patients with type 1 SMA when administered before six months of age at the dose of 1.1 × 10 ^14^ viral genomes [vg]/kg. 92 93 In these phase 3 trials, a high proportion of treated patients achieved important motor milestones rarely observed in the natural course of the disease. Between 44% to 59% of treated patients could sit unsupported at 18 months, compared to none of the 23 patients with similar baseline characteristics from the Pediatric Neuromuscular Clinical Research (PNCR) natural history study cohort. Furthermore, between 91% to 97% of the infants treated with onasemnogene abeparvovec were alive and free from mechanical ventilation at 14 months of age, in contrast to only 26% of patients from the PNCR study cohort. 92 93 Notably, the maximum weight of patients included in these clinical trials was 8.4 kg. 90 92 93

A posthoc analysis of pooled data from one phase 1 (START) and two phase 3 (STR1VE-US, STR1VE-EU) studies evaluated the bulbar function of infants with SMA type 1 after receiving gene replacement therapy. 94 After 18 months (STR1VE-US and STR1VE-EU) or 24 months (START) post-infusion, 92% of patients had a normal swallow, 75% achieved full oral nutrition, and 95% met the communication endpoint. 94

After the approval of onasemnogene abeparvovec, several real-world studies have been conducted in different countries to assess its effectiveness and safety in broader populations beyond the initial clinical trials. These real-world studies have confirmed the efficacy of the gene replacement therapy in an expanded age range of patients eligible for treatment, including those up to two years old and also patients with three copies of *SMN2* , regardless of the type of SMA. 95 96 97 98 99 100 101 102 103 104 105 106 107 108 109 Additionally, some studies have evaluated the use of onasemnogene abeparvovec in patients who had previously been treated with other specific therapies, such as nusinersen or risdiplam. 102 103 105 106 108

### Pre-symptomatic SMA-5q treatment with gene replacement therapy

The SPR1NT (CL-304) phase 3 study has provided crucial evidence on the efficacy of gene therapy in pre-symptomatic children with SMA-5q who received treatment at a very early age, specifically ≤ 6 weeks old. 110 111 The study enrolled 29 pre-symptomatic children with a confirmed genetic diagnosis of SMA-5q, either with 2 or 3 *SMN2* gene copies. The study’s results showed significant positive outcomes in motor function for both groups of patients. In children with three *SMN2* copies, all 15 participants stood independently before 24 months, within the expected developmental window. 111 Additionally, 14 of the 15 children walked independently within the expected developmental window, and 10 (67%) maintained body weight (≥3rd WHO percentile) without requiring feeding support through 24 months. For the 14 enrolled infants with two *SMN2* copies, all of them achieved the ability to sit independently for ≥ 30 seconds at any visit before 18 months of age. 110 Furthermore, 13 of these children maintained body weight (≥ 3rd WHO percentile), indicating adequate nutritional status. Importantly, all patients with two or three *SMN2* copies survived without permanent ventilation at 14 months, and none of the children required nutritional or respiratory support. At 18 months (children with two copies of *SMN2* ) and 24 months (children with three copies of *SMN2* ), all children swallowed normally and achieved full oral nutrition. 112

Real-world studies conducted in various countries, particularly those linked to expanded newborn screening (NBS) programs, have consistently shown that early initiation of gene replacement therapy in pre-symptomatic infants with SMA-5q results in remarkable developmental progress, with children achieving motor milestones within the expected developmental timeframe. 108 113 114 115 Furthermore, cost-effectiveness studies, particularly in countries like Australia, have shown that implementing neonatal screening for SMA-5q, coupled with early gene replacement therapy treatment, leads to reduced costs or remains within the cost-effectiveness threshold considered by these countries in the long term. 116

### Safety of gene replacement therapy with onasemnogene abeparvovec

The safety profile of onasemnogene abeparvovec has been extensively studied and monitored in preclinical studies, clinical trials, real-world studies, registries, and expanded access programs involving hundreds of patients with SMA-5q. Adverse events reported in preclinical studies included cardiac and hepatic effects with systemic administration and effects on dorsal root ganglion neurons with intrathecal administration. 117

In human clinical trials and real-world settings, treatment-related severe adverse events have been reported in just over 10% of cases, with the most common being liver function abnormalities and fever. 92 109 118 Other important adverse event includes thrombocytopenia. It is essential to note that while most patients experience manageable adverse events, there have been reports of rare but severe adverse events. Fatal cases of thrombotic microangiopathy and acute liver failure have been reported, 119 120 as well as potentially fatal conditions such as hemophagocytic syndrome and necrotizing enterocolitis. 121 122

### AAV9 titer measurement

AAV9 is used as a vector to deliver the therapeutic gene in onasemnogene abeparvovec. However, if a patient has pre-existing antibodies against AAV9 in their blood, these antibodies can neutralize the viral vector, preventing it from effectively delivering the therapeutic gene to the target cells. To maximize the chances of successful gene therapy, patients need to have AAV9 antibody titers below a certain threshold, typically no greater than 1:50. Day et al (2021) showed that 7.7% of patients with SMA-5q have pre-existing titers of anti-AAV9 antibodies in their blood above the safety threshold. However, it's essential to note that some patients may have transient or decreasing antibody titers over time, which may still make them eligible for treatment after reevaluation. 123

### Prospects

The STRONG study is a clinical trial that evaluated the safety, tolerability, and efficacy of an intrathecal single dose of onasemnogene abeparvovec in non-ambulatory patients with SMA-5q who have three copies of the *SMN2* gene and aged 6 to under 60 months. 124 This study focused on assessing the treatment's effects on sitting ability in these patients. During the study, all patients experienced one or more treatment-emergent adverse events, but only one was considered serious and related to treatment, involving serum transaminase elevations. Regarding efficacy, in the younger group (6 to under 24 months) treated with the medium dose, one out of thirteen patients (7.7%) achieved independent standing. For the older group (24 to under 60 months) receiving the medium dose, there was a significant improvement in the change from baseline in the HFMSE compared with the SMA-5q historic control population at month 12. 124 Further research is ongoing to explore the use of lower intrathecal doses of onasemnogene abeparvovec in patients aged 2 to under 18 years in a randomized multicenter controlled clinical trial (NCT05089656).

### Follow-up of SMA-5q patients undergoing drug treatment

The follow-up of SMA-5q patients who are treated with disease-modifying therapies is essential, both as part of the proactive management of these patients and to assess the response to medications ( Chart 5 ). Multidisciplinary care combined with new therapies remains imperative, necessitating an approach focused on each patient's clinical status and current needs to optimize quality of life and motor, respiratory, and bulbar function. Age, *SMN2* copy number, and baseline motor function are important determinants when setting goals to be achieved with the treatment. 125

**Chart 5:** Follow-up recommendations for patients with SMA-5q undergoing drug treatment

Multidisciplinary care and disease-modifying therapies	The treatment of patients with SMA-5q should involve a combination of multidisciplinary care and disease-modifying therapies. This approach ensures comprehensive management and optimal outcomes for the patients
Key domains for monitoring	The follow-up of patients should focus on monitoring motor function, respiratory function, bulbar function (speech and swallowing), and nutrition. Regular assessments of these domains are essential to track the patient's progress and response to treatment
Use of clinical scales and assessment instruments	Clinical scales and other validated assessment instruments should be applied during follow-up to evaluate the response to drug therapies accurately.
Effective treatment outcomes	Treatment should be considered effective when there is improvement or stabilization in one or more of the monitored domains (motor, respiratory, nutritional, and bulbar)
Follow-up intervals	The follow-up intervals for patients with SMA-5q depend on the severity of their clinical condition. Patients with SMA type 1 should be followed up every 3-6 months on average, while patients with SMA types 2 and 3 should be followed up every 6 months on average
Discontinuation criteria for type 1 SMA	For children with SMA type 1, treatment discontinuation or replacement may be considered if there is a loss or lack of gain in motor and/or respiratory functions within an average period of 6 months of treatment. Motor and/or respiratory stabilization can also be considered a satisfactory outcome for those with a more chronic condition
Discontinuation criteria for types 2 and 3 SMA	For patients with SMA types 2 and 3, treatment discontinuation or replacement may be considered if there is functional loss or lack of disease stabilization detected within an average period of 12 months of follow-up
Switching therapy	Consider switching from one therapy to another if difficulties arise in applying intrathecal nusinersen due to worsening scoliosis, and if the patient experiences a treatment failure or lack of response to the current therapy
Combined therapies	The combined use of two or more modifying therapies in patients with SMA-5q has limited evidence in the medical literature regarding additional efficacy and safety. Thus, as of now, combination therapy for AME-5q is not recommended due to the limited data available

A systematic follow-up with validated instruments is crucial to assess the response to treatment. Pivotal clinical trials showed the importance of primary and secondary endpoints to evaluate the efficacy of the new therapies. 24 41 73 77 90 Specific instruments were used, but the evaluations were variable and dependent on the clinical phenotype of SMA.

Considering the pivotal drug studies, the main instruments were motor function scales, motor achievements, survival and ventilation-free, bulbar function, nutritional state, and electrophysiological studies. 24 41 73 77 90 Some biomarkers, such as neurofilaments, SMN protein measurement, and neurophysiological assessments (Compound Muscle Action Potential - CMAP), have been included in the follow-up of the patients in different studies. 24 41 73 77 90 However, these biomarkers still need more investigations to have their use recommended in clinical practice.

### Real-world assessment of therapeutic efficacy

Several domains must be evaluated, considering that the treatment is effective if the improvement is demonstrated in one or more of these domains: motor function, motor milestones, respiratory function, bulbar function (speech and swallowing), and nutritional data. 126

The SMArtCARE project is a platform to collect real-life outcomes data of patients with SMA in Germany, Austria, and Switzerland. It proposes standardized instruments to be used according to age and functional motor capacity ( Table 2 ). 127 In children under 12 years of age, the HINE should be used to evaluate motor milestones. 127

**Table 2:** SMArtCARE recommendations for motor evaluation of SMA-5q patients 127

CHOP INTEND	HFMSE	RULM	6MWT
All children <2 years old All patients > 2 years old without the ability to sit	All patients >2 years old with the ability to sit If CHOP INTEND score > 50: CHOP INTEND and HFSME If CHOP INTEND score > 60: HFMSE instead of CHOP INTEND	All patients > 2 years old with the ability to sit (in a wheelchair)	All ambulant patients > 3 years old

Abbreviations: 6MWT, 6-minutes walking test; CHOP INTEND, Children's Hospital of Philadelphia Infant Test of Neuromuscular Disorders; HFMSE, Hammersmith Functional Motor Scale Expanded; RULM, Revised Upper Limb Module.

Different motor scales should be used in combination as each of them contributes to detecting possible changes in different groups of patients. While the 6MWT can only be performed in ambulant patients, the Revised Upper Limb Module scale (RULM) is more appropriate for weaker patients as it often reaches ceiling scores in stronger ambulant type 3 SMA patients. 52 128 Considering the reality in Brazil, where there is difficulty in many centers to perform the 6MWT, this test can be replaced by timed tests to assess the motor function of ambulant patients, such as the time needed to walk 10 meters, rise from the floor, and climb steps. 129

From a respiratory perspective, therapies may improve the progression of respiratory impairment. The strongest indicators of therapeutic response are not requiring invasive ventilation or respiratory support (especially before the age of 2 years), a reduction in the hours of non-invasive ventilation (NIV), or fewer pulmonary infections (or hospitalizations for such infections). 128 Disease progression could be considered if the patient requires invasive ventilation or NIV for more than 16 hours a day for more than 21 continuous days in the absence of an acute reversible event or spends more hours on ventilation and has no reduction in the number of respiratory infections and hospitalizations. 128 130 From the age of 6 years, peak cough flow (PCF) and spirometry studies should be performed, including forced vital capacity (FVC) and peak expiratory flow (PEF). However, this may be attempted from 4 to 5 years in collaborative patients ( Table 3 ). 131 The use of polysomnography and nocturnal oximetry as markers of therapeutic response in SMA type 1 is still unclear. 132 133

**Table 3:** Global assessment of SMA-5q patients 32 126 128

	Assessment of non-sitters	Assessment of sitters	Assessment of walkers
**Motor function**	HINE-2, CHOP INTEND (or CHOP ATEND for adults), RULM	HINE-2, CHOP INTEND (or CHOP ATEND for adults), HFSME, RULM, MFM32	HFSME, 6MWT, MFM32, RULM, Timed-test (time needed to walk 10 m, arise from the floor, and climb steps)
**Respiratory function**	Need of NIV or IV, changes in time required for NIV, number of recurrent pulmonary infections, hospitalizations (number of days a year)	Need of NIV or IV, changes in time required for NIV, number of recurrent pulmonary infections, hospitalizations (number of days a year) PCF Spirometry (≥ 6 years old): FVC, PEF	Spirometry (≥ 6 years old): FVC, PEF PCF
**Bulbar function**	Need of nasogastric tube or gastrostomy; Videofluoroscopic swallow study or clinical assessment of the risk of aspiration	Need of nasogastric tube or gastrostomy Videofluoroscopic swallow study or clinical assessment of the risk of aspiration	
**Nutritional status**	Clinical evaluation: Weight, height BMI Percentile > 3 Z-score > -2	Clinical evaluation: Weight, height BMI Percentile > 3 Z-score > -2	Clinical evaluation: Weight, height BMI Percentile > 3 Z-score > -2

Abbreviations: 6MWT, 6-minutes walking test; BMI, Body mass index; CHOP ATEND, Children's Hospital of Philadelphia’s Adult Test of Neuromuscular Disorders; CHOP INTEND, Children's Hospital of Philadelphia Infant Test of Neuromuscular Disorders; FVC, forced vital capacity; HFMSE, Hammersmith Functional Motor Scale Expanded; HINE-2, Hammersmith Infant Neurological Examination 2; IV, invasive ventilation; MFM32, Motor Function Measure 32; NIV, non-invasive ventilation; PCF, Peak cough flow; PEF, peak expiratory flow; RULM, Revised Upper Limb Module.

Consistent with the literature, there is poor availability of outcome measures for bulbar function. 126 Therefore, we suggest that the evaluation of therapeutic efficacy in this domain be done through the need for a nasogastric tube or gastrostomy, video fluoroscopy swallow study, or clinical assessment of the risk of aspiration. 131

Fatigue is a complex issue but a frequent complaint of SMA patients, especially in types 2 and 3. There are different instruments based on fatigue classification to evaluate fatigue: physical or observational. 134 However, it is still considered a challenge, and no specific protocol exists to evaluate this manifestation.

The poor availability of outcome measures that assess important domains of quality of life, fine motor skills, and endurance (particularly in non-sitter and sitter populations) led to the routine assessment of these domains not being included in this consensus. 126 135 The patient's perception of satisfaction, response to treatment, and adverse effects are important, but there are no outcome measures for these domains. 136

The frequency with which patients should be reassessed needs to be better defined in the literature and varies greatly according to the practice of different care centers in the world. 52 However, there is consensus that individuals with SMA type 1 should be reassessed at least once every 3 to 6 months and individuals with SMA types 2 and 3 at least once every 6 to 12 months. 52 126 128 In Brazil, the clinical protocol and therapeutic guideline for treating patients with SMA types 1 and 2 require that patients undergoing treatment need to be followed in reference centers and have motor, respiratory, and nutritional function reassessed every six months; and only one motor scale is required ( Table 3 ). 137

### Criteria for interruption and switch of drug treatment

For evaluation of treatment efficacy, SMA type 1 patients should be treated over at least six months, and SMA type 2 and 3 over 12 months. 27 48 128 Due to the progressive nature of SMA-5q, improvement or stabilization of functions in patients over two years is considered a therapeutic response. 137

Publications from different countries showed different criteria for interrupting or switching drug treatment, considering their social and economic reality. In Brazil, according to the government technical protocol, the drug treatment should be stopped if there are no clinical benefits associated with the treatment, if there is an evolution to permanent invasive ventilation for 24 hours a day for a period longer than 90 days, or if the patient develops brain injury. 137 The discontinuation of the treatment should also be done if there is hypersensitivity or severe adverse reaction to the drug, and in these situations, another drug can be used.

The drug's switch is also a controversial issue. In some countries, especially when there is insurance coverage, the switch from one drug to another can be more widely considered, as in situations of partial response to one drug (for example, improvement in motor domain but not bulbar function) and based on family preferences. 138 In Brazil, the government technical protocol suggests the drug's switch based on medical decisions, considering: a) Nusinersen to risdiplam: occurrence of serious adverse events, difficulties in the administration of the medication including scoliosis or contractures, in case of appearance of spinal or cerebral disease, necessity of catheter or ventriculoperitoneal shunt or in the treatment ineffectiveness (considering motor function after 12 months of treatment); b) Risdiplam to nusinersen: occurrence of serious adverse events, during pregnancy or breastfeeding period, in the treatment ineffectiveness (considering motor function after 12 months of treatment. 137

### Combination or sequential therapy

Until now, no evidence exists that combination or sequential therapy offers meaningful clinical benefit for patients with SMA-5q. 139 The impact of combination therapy on patient outcomes and safety is unknown and is being evaluated in clinical trials. 140 Mirea et al (2021) 141 based on the observation of two groups of SMA type 1 patients; one group received two therapies, nusisersen and gene replacement therapy, and the control group received only nusinersen, showed that patients who received both therapies, compared to the monotherapy cohort, had the same motor function trajectory. They concluded that early treatment is more critical than combined treatment. 141

### Genetic counseling

Chart 6 provides a summary of genetic counseling recommendations for SMA-5q. SMA-5q is an autosomal recessive disorder mostly associated with a homozygous deletion in the *SMN1* gene in approximately 96% of the cases, whose carrier frequency ranges from 1/35 to 1/60 in most populations. 142 However, approximately 4% of patients have a compound heterozygous condition, including a deletion in one *SMN1* gene and a single nucleotide variation (SNV) in the other gene. Patients may also develop the disease due to small-scale pathogenic variations in both alleles. When the affected child has a homozygous pathogenic variant (common deletion or SNV) in the *SMN1* gene, and both parents have one normal *SMN1* gene and one allele with a pathogenic variant, then the risk of a new affected child is 25%. At the same time, 50% of the descendants will be carriers.

**Chart 6:** Genetic counseling recommendations for SMA-5q

Genetic counseling for parents of SMA patients	A qualified professional should provide genetic counseling to the parents of patients diagnosed with SMA-5q. The purpose of counseling is to inform the parents about the genetic basis of the condition, the risk of recurrence in future pregnancies, and available testing options. Genetic testing, such as MLPA and/or gene sequencing, can be performed to identify heterozygous variants in the *SMN1* gene in both parents to understand the risk of SMA-5q in future offspring
Genetic counseling for close relatives of SMA patients	Genetic counseling should also be offered to close relatives of SMA patients who may be at risk of being carriers of the *SMN1* gene mutation. Identifying carriers within the family is important for understanding the risk of SMA-5q in other family members and providing appropriate counseling and testing options
Genetic counseling for potential partners of adult SMA patients	For adult patients with SMA-5q, genetic counseling should be provided to potential partners. This counseling aims to discuss the genetic implications of SMA-5q and the risk of having children with SMA-5q if both partners are carriers of the *SMN1* gene mutation. MLPA testing and/or gene sequencing can be offered to potential partners to assess carrier status

A more complicated situation happens when one of the parents has two intact *SMN1* genes, a condition seen in about 6% of the parents. 142 There are three possible explanations: 1) *de novo* deletion occurs in about 2% of the cases, most are of paternal origin, and the risk of recurrence is rare; 2) germline mosaicism is another possible explanation, but its diagnosis is complicated and 3) approximately 4-8% of the carriers have a heterozygous condition with two *SMN1* genes in *cis* in one chromosome and a deleted *SMN1* gene in the other allele. 143 144 145 Recognizing these diverse possibilities is very important to have proper genetic counseling.

The presence of a deletion in the *SMN1* gene is detected by quantitative methods (MLPA or qPCR), while the detection of SIVs demands sequencing of the entire coding region of the gene. 144 Other genomic variations demand a family study with haplotype analysis. 144

In principle, genetic counseling and genetic testing should be offered to all relatives with a carrier risk ≥ 1/8, 145 to the parents of SMA patients with the potential to have new children and to all adult SMA patients aiming to have children ( Chart 6 ).

## DISCUSSION

The consensus recommendations provided by Brazilian physicians specializing in treating patients with SMA-5q highlight essential aspects of genetic diagnosis, genetic counseling, and disease-modifying therapies for patients with SMA-5q.

The genetic confirmation of patients with SMA-5q is essential, particularly in demonstrating pathogenic variants in both alleles of the *SMN1* gene ( Chart 1 ). 145 Genetic counseling is crucial for understanding the genetic basis of the condition, the risk of recurrence in future pregnancies and guiding treatment decisions ( Chart 6 ). 145 In addition to genetic counseling, informing the parents about the diagnosis and prognosis of the disease should also be part of the patient's medical consultation.

Regardless of the therapy chosen for treatment, this should be started as early as possible, seeking to preserve the remaining motoneurons as much as possible. The ideal scenario is to start therapy in the pre-symptomatic phase of the disease or when the child is in a very early stage. 146 147 Chronic patients with long-term illness and severe motor and respiratory impairment have less favorable outcomes for any therapies used. 148 So, the next step to obtain better outcomes in the era of new therapies for SMA-5q is the implementation of newborn screening in our country.

For patients in pre-symptomatic stages of the disease, the Brazilian Public Health System (SUS) offers treatment only for babies with up to three copies of the *SMN2* gene. 137 However, there is consensus in the medical literature to indicate treatment for pre-symptomatic patients with up to four copies of the *SMN2* gene. 149 In addition, recent work confirmed the variability of phenotypes in untreated patients with four copies of *SMN2* , ranging from type 2 to type 4, and an overall reduction of functional scores with increasing age. 150 These findings indicate the importance of treating these patients earlier.

Currently, in Brazil, two *SMN2* gene exon 7 splicing modifier therapies, nusinersen and risdiplam, are available through the SUS for pre-symptomatic patients, or children with SMA type 1 (onset before 6 months) or type 2 (onset between 6 to 18 months, and only those older than 12 years if they can sit without support and also have preserved upper limb function). All treated patients must have three or fewer copies of *SMN2* . 137 Treatment is not yet available in the SUS for those with SMA types 3 or 4, any SMA patient on permanent mechanical ventilation, or any SMA patients with four or more copies of *SMN2* .

Nusinersen is an intrathecal drug in which the patient needs to receive therapy in a hospital environment. The most significant limitation of using the therapy is cases with a complex spine (severe scoliosis or previous spine arthrodesis). Such situations do not prevent the intrathecal administration of the therapy; however, it requires special care during its administration, especially with the use of imaging techniques and by experienced personnel. 151 If one of the doses is missed, the patient should receive the next dose as soon as possible. The subsequent dose may then be maintained according to the previously defined schedule. Detailed advice based on clinical trial analysis and pharmacokinetic studies is available 152 and is reassuring that only prolonged omission of eight months or more should require additional dosing.

Risdiplam is an oral and daily administration treatment. According to the drug insert approved by ANVISA, there is a need for the suspension of the drug to be carried out in a hospital environment and subsequently supplied to the patient already in liquid form. 153 Another important recommendation is the need to keep the medicine refrigerated and protected from light. ANVISA approved risdiplam for all forms of SMA from 16 days of life.

Gene replacement therapy with onasemnogene abeparvovec was incorporated into the SUS for pre-symptomatic babies and type 1 SMA up to six months of age without permanent ventilation. It is a single intravenous dose treatment, which must be done at the hospital level. The centers must be previously qualified to administer the therapy and must be capable of managing complications of the therapy. Although gene replacement therapy is administered in a single dose, there is a need for concomitant administration of corticosteroids due to the risk of immune-mediated reactions. The physicians in charge should monitor the patient with attention to the management of corticosteroid therapy and the emergence of possible adverse events. 140

It should be noted that the greater the child's body weight, the greater the risk of adverse events since the dose of the vector administered is also higher. 98 109 154 Thus, the safety of gene replacement therapy in older children is not well defined in the medical literature, nor is the duration of treatment effect. However, long-term efficacy and safety for at least five years have been demonstrated. 91 As discussed before, two complications are greatly to be feared. Thrombotic microangiopathy occurs rarely but can be fatal. 98 109 It occurs in the first weeks after replacement gene therapy and must be promptly treated with plasmapheresis or eculizumab (the latter is unavailable in the SUS). The other potentially severe complication is hepatic injury, with elevated liver enzymes progressing to liver failure and death. The care of this complication is done with corticosteroid therapy. 98 109 The use of corticosteroids can bring some problems to patients, such as weight gain and immunosuppression. 155 So, patients eligible for gene replacement therapy need to be closely followed from the moment of the treatment indication in centers with a multidisciplinary team and infrastructure to manage the adverse events related to the medication.

Regardless of the treatment instituted, these patients must be followed up indefinitely. Modifying therapies should be monitored to detect adverse events and unexpected manifestations and evaluate short- and long-term effectiveness. Regardless of the type of therapy instituted, the follow-up objectives include maintaining multidisciplinary care, such as motor, respiratory, bulbar, nutritional, and osteoskeletal care. 128 Specific motor scales should be applied regularly to assess the gains from therapies. 127 The baseline of each patient needs to be detailed by the multidisciplinary team before starting any drug. Based on these data, the family expectations can be discussed and aligned with the patient’s condition. For cases in which there is a therapeutic failure, the physician must either suspend the treatment or replace it with another treatment. This consensus does not currently recommend the concomitant administration of two or more therapies.

The therapies are expected to provide functional and score gains on the scales for younger children, up to two years of age because these children are still in the development phase. In contrast, stabilization of the motor, bulbar, and respiratory conditions is expected in older children, especially after two years of age.

In conclusion, this consensus emphasizes the importance of individualized treatment plans based on the patient's age, disease severity, and response to therapy. Regular monitoring and evaluation are vital to optimize treatment outcomes for patients with SMA-5q. The expertise of healthcare professionals and qualified specialists is crucial in effectively managing SMA and its therapies. The involvement of experienced specialists and the emphasis on evidence-based recommendations enhance the reliability and applicability of the consensus in clinical practice. A robust newborn screening program for SMA-5q in Brazil will be the ideal scenario for better outcomes.
